# Soft Approximations and Uni-Int Decision Making

**DOI:** 10.1155/2014/327408

**Published:** 2014-03-04

**Authors:** Athar Kharal

**Affiliations:** National University of Sciences and Technology (NUST), Islamabad 44000, Pakistan

## Abstract

Notions of core, support, and inversion of a soft set have been defined and studied. Soft approximations are soft sets developed through core and support and are used for granulating the soft space. Membership structure of a soft set has been probed in and many interesting properties are presented. We present a new conjecture to solve an optimum choice problem. Our Example 31 presents a case where the new conjecture solves the problem correctly.

## 1. Introduction

Applications of soft set theory in many real life problems are now catching momentum. Molodtsov (1999) [1] successfully applied the soft set theory into several directions, such as smoothness of functions, Riemann integration, Perron integration, theory of probability, and theory of measurement.

Soft sets have been used extensively for different decision making problems. Maji et al. (2002) [2] gave first practical application of soft sets in decision making problems. It is based on the notion of knowledge reduction in rough set theory. Chen et al. (2005) [3] made some improvements in the work of Maji et al. (2002) and introduced a new kind of parametrization reduction [3] of soft sets. Chen's reduction of parameters of a soft set is designed to offer minimal subset of the conditional parameters set while preserving the object(s) of optimal choice. In [4], the authors based their algorithm for natural texture classification on soft sets. This algorithm has a low computational complexity when compared to a Bayes technique based method for texture classification. In [5] Rose et al. (2010) applied the theory of soft set to data analysis and decision support systems again. By applying the concept of cooccurrence of parameters in an object and its support on the Boolean-valued information based on soft set theory, they propose an alternative technique termed as maximal supported sets reduct. Luo and Shen (2010) [6] proposed a suitable credit risk evaluation method using soft set theory. Their method integrates the operating characteristics with the credit risk evaluation method so that loan officers can make loan decision more accurate using the available soft information. Herawan and Deris (2010) [7] have also tackled a decision making problem of patients suspected influenza. The work is based on maximal supported objects by parameters. At this stage of the research, results are presented and discussed from a qualitative point of view against recent soft decision making techniques through an artificial dataset.

Organization of the paper is as follows. In Section 2, we first introduce the simple notions of core and support of a soft set.  Section 3 comprises three different notions of membership structure of a soft set. We study many of the fundamental properties of soft membership therein and also give counterexamples where it is imperative.  Section 4 introduces and studies the notions of upper and lower soft approximations. These approximations are shown to have links with upper and lower approximations of a subset of universe introduced by Feng et al. in [8]. Whereas the approximations of Feng et al. provide a way to granulate the universe of discourse, soft approximations granulate the soft space itself.

## 2. Preliminary Notions

Throughout this paper, *X* refers to an initial universe, and *E* is a set of parameters. Molodtsov (1999) [1] defined a soft set as a pair (*F*, *A*), where *F* is a mapping given by *F* : *A* → *P*(*X*) and *P*(*X*) is the power set of *X*. The pair (*X*, *E*), called a soft space [9, 10], is the collection of all soft sets on *X* with attributes from *E*. We shall use the words “parameter” and “attribute” interchangeably. In the sequel the cardinality of a set *A* is denoted as ||*A*||.

**Definition 1 (see [11])**

Soft union of two soft sets (*F*, *A*) and (*G*, *B*) in a soft space (*X*, *E*) is a soft set (*H*, *C*), where *C* = *A* ∪ *B* and, for all *e* ∈ *C*,

(1) $$\begin{array}{c} H\left(e\right)=\{\begin{array}{cc} F\left(e\right), & \text{if}\;\;e\in A-B,\\ G\left(e\right), & \text{if}\;\;e\in B-A,\\ F\left(e\right)\cup G\left(e\right), & \text{if}\;\;e\in A\cap B, \end{array} \end{array}$$

and is written as $\left(F,A)\;\tilde{\cup }\;(G,B)=(H,C\right)$.

**Definition 2 (see [12])**

Extended soft intersection of two soft sets (*F*, *A*) and (*G*, *B*) in a soft space (*X*, *E*), is a soft set (*H*, *C*), where *C* = *A* ∪ *B*, and for all *e* ∈ *C*,

(2) $$\begin{array}{c} H\left(\varepsilon \right)=\{\begin{array}{cc} F\left(e\right), & \text{if}\;\;e\in A-B,\\ G\left(e\right), & \text{if}\;\;e\in B-A,\\ F\left(e\right)\cap G\left(e\right), & \text{if}\;\;e\in A\cap B, \end{array} \end{array}$$

and is written as $\left(F,A)\;\tilde{\cap }\;(G,B)=(H,C\right)$. Hereafter this type of intersection will be called soft intersection in this paper.

**Definition 3 (see [12])**

Restricted soft union of two soft sets (*F*, *A*) and (*G*, *B*) in a soft space (*X*, *E*), such that *A*∩*B* ≠ *ϕ*, is a soft set (*H*, *C*), where *C* = *A*∩*B* and for all *e* ∈ *C*,

(3) $$\begin{array}{c} H\left(e\right)=F\left(e\right)\cup G\left(e\right). \end{array}$$

This is written as (*F*, *A*)⋓(*G*, *B*) = (*H*, *C*).

**Definition 4 (see [12])**

Restricted soft intersection of two soft sets (*F*, *A*) and (*G*, *B*) in a soft space (*X*, *E*), such that *A*∩*B* ≠ *ϕ*, is a soft set (*H*, *C*), where *C* = *A*∩*B* and, for all *e* ∈ *C*,

(4) $$\begin{array}{c} H\left(e\right)=F\left(e\right)\cap G\left(e\right). \end{array}$$

This is written as (*F*, *A*)⋒(*G*, *B*) = (*H*, *C*).

**Definition 5 (see [13])**

For two soft sets (*F*, *A*) and (*G*, *B*) in a soft space (*X*, *E*), we say that (*F*, *A*) is a soft subset of (*G*, *B*) if

- *A*⊆*B*,
- for all *e* ∈ *A*, *F*(*e*)⊆*G*(*e*).

In the sequel, we shall write $\left(F,A)\;\tilde{\subseteq }\;(G,B\right)$ to denote the inclusion in the sense of Pei and Miao.

**Definition 6 (see [12])**

Let (*X*, *E*) be a soft space and *A* ⊂ *E*.

1. (*F*, *A*) is said to be a relative null soft set denoted by ${\tilde{\Phi }}_{A}$ if *F*(*e*) = *ϕ*, for all *e* ∈ *A*.
2. (*G*, *A*) is said to be a relative whole soft set denoted by ${\tilde{X}}_{A}$ if *F*(*e*) = *X*, for all *e* ∈ *A*.

The relative null (resp., whole) soft set ${\tilde{\Phi }}_{E}$ (resp., ${\tilde{X}}_{E}$) is called null (resp., absolute) soft set and is denoted by $\tilde{\Phi }$ (resp., $\tilde{X}$).

**Definition 7 (see [11])**

Let *E* = {*e*_1_, *e*_2_, *e*_3_,…, *e*_*n*_} be a set of parameters. The NOT set of *E* denoted by ⌉*E* is defined by ⌉*E* = (¬*e*_1_, ¬*e*_2_, ¬*e*_3_,…, ¬*e*_*n*_}, where ¬*e*_*i*_ = not *e*_*i*_, for all *i*. (It may be noted that ⌉ and ¬ are different operators.)

**Definition 8**

For a soft set (*F*, *A*) = {*e* = *F*(*e*) | *e* ∈ *A*} in a soft space (*X*, *E*) the not of (*F*, *A*) is denoted and defined as

(5) $$\begin{array}{c} {\left(F,A\right)}^{\neg }=\left\{e=F\left(e\right)\;|\;e\in \rceil A\right\}. \end{array}$$

**Definition 9 (see [12])**

For a soft set (*F*, *A*) = {*e* = *F*(*e*) | *e* ∈ *A*} in a soft space (*X*, *E*) the relative complement of (*F*, *A*) is denoted and defined as

(6) $$\begin{array}{c} {\left(F,A\right)}^{c}=\left\{e={\left(F\left(e\right)\right)}^{c}\;|\;e\in A\right\}. \end{array}$$

In this paper, hereafter, “relative complement” will be called simply a complement of soft set (*F*, *A*).

## 3. Core, Support, and Membership of a Soft Set

**Definition 10**

For a soft set (*F*, *A*) in soft space (*X*, *E*) core and support of (*F*, *A*) are denoted and defined as $\underline{FA}={⋂}_{e\in A}^{}F\left(e\right)$ and $\bar{FA}={⋃}_{e\in A}^{}F\left(e\right)$, respectively. Clearly $\underline{FA}$, $\bar{FA}\subseteq X$.

Few of the fundamental properties of these notions are given below.

**Proposition 11**

For soft sets (*F*, *A*) and (*G*, *B*) in a soft space (*X*, *E*), we have

1. $\underline{FA}\subseteq \bar{FA}$,
2. $\bar{\left(F,A\right)\;\tilde{\cup }\;\left(G,B\right)}=\bar{FA}\cup \bar{GB}$,
3. $\bar{\left(F,A\right)⋓\left(G,B\right)}\subseteq \bar{FA}\cup \bar{GB}$,
4. $\bar{\left(F,A\right)⋒\left(G,B\right)}\subseteq \bar{FA}\cap \bar{GB}$,
5. $\underline{FA}\cup \underline{GB}\subseteq \underline{\left(F,A\right)⋓\left(G,B\right)}$,
6. $\underline{FA}\cap \underline{GB}\subseteq \underline{\left(F,A\right)⋒\left(G,B\right)}$,
7. $\underline{\left(F,A\right)\;\tilde{\cap }\;\left(G,B\right)}=\underline{FA}\cap \underline{GB}$,
8. ${\left(\bar{FA}\right)}^{c}\subseteq \underline{{\left(F,A\right)}^{c}}$,
9. $\underline{{\left(F,A\right)}^{c}}\subseteq {\left(\underline{FA}\right)}^{c}$,
10. $\left(F,A\right)\;\tilde{\subseteq }\;\left(G,B\right)\Rightarrow \bar{FA}\subseteq \bar{GB}$.

**Proof**

(3)  $x\in \bar{\left(F,A\right)⋓\left(G,B\right)}$ implies there exists *ε* ∈ *A*∩*B* such that *x* ∈ *F*(*ε*) or *x* ∈ *G*(*ε*); that is, $x\in \bar{FA}$ or $x\in \bar{GB}$, by definition of support. Equivalently $x\in \bar{FA}\cup \bar{GB}$.

(4)  $x\in \bar{\left(F,A\right)⋒\left(G,B\right)}$, and there exists *ε* ∈ *A*∩*B* such that *x* ∈ *F*(*ε*) and *x* ∈ *G*(*ε*); hence $x\in \bar{FA}$ and $x\in \bar{GB}$. Equivalently $x\in \bar{FA}\cap \bar{GB}$.

(5) Note that *A*∩*B* ≠ *ϕ*, as otherwise restricted union is not defined. For $x\in \underline{FA}\cup \underline{GB}$, there are two cases: if $x\in \underline{FA}$ and $x\in \underline{GB}$, both, we have *x* ∈ *F*(*ε*) for all *ε* ∈ *A* and *x* ∈ *G*(*δ*) for all *δ* ∈ *B*. Hence for all *γ* ∈ *A*∩*B*,  *x* ∈ *F*(*γ*), and *x* ∈ *G*(*γ*),  $x\in \underline{\left(F,A\right)⋓\left(G,B\right)}$.

In the second case, suppose $x\in \underline{FA}$ only. Then

(7) $$\begin{array}{c} \forall \varepsilon \in A,\;x\in F\left(\varepsilon \right) \end{array}$$

and as *A*∩*B* ≠ *ϕ*, let *γ* ∈ *A*∩*B*. By (7) *x* ∈ *F*(*γ*), this implies *x* ∈ *F*(*γ*) ∪ *G*(*γ*) for all *γ* ∈ *A*∩*B*. Equivalently $x\in \underline{\left(F,A\right)⋓\left(G,B\right)}$.

(6)  $x\in \underline{FA}\cap \underline{GB}$, which implies

(8) $$\begin{array}{c} \forall \varepsilon \in A,\;x\in F\left(\varepsilon \right), \end{array}$$

(9) $$\begin{array}{c} \forall \delta \in B,\;x\in G\left(\delta \right). \end{array}$$

For *γ* ∈ *A*∩*B* ≠ *ϕ*, we have *x* ∈ *F*(*γ*) and *x* ∈ *G*(*γ*), by (8) and (9), respectively. That is for all *γ* ∈ *A*∩*B*,  *x* ∈ *F*(*ε*)∩*G*(*ε*). This proves that $x\in \underline{\left(F,A\right)⋓\left(G,B\right)}$.

(7)  $x\in \underline{FA}\cap \underline{GB}$, which implies

(10) $$\begin{array}{c} \forall \varepsilon \in A,\;x\in F\left(\varepsilon \right), \end{array}$$

(11) $$\begin{array}{c} \forall \delta \in B,\;x\in G\left(\delta \right). \end{array}$$

Let *γ* ∈ *A* ∪ *B*. Then

(12) $$\begin{array}{c} x\in F\left(\gamma \right)\;\text{if}\;\;\gamma \in A\setminus B\;\;\text{by}\;\;\left(10\right),\\ x\in G\left(\gamma \right)\;\text{if}\;\;\gamma \in B\setminus A\;\;\text{by}\;\;\left(11\right),\\ x\in F\left(\gamma \right),\;x\in G\left(\gamma \right)\;\text{if}\;\;\gamma \in A\cap B\;\;\text{by}\;\;\left(10\right)\;\text{and}\;\left(11\right), \end{array}$$

which implies $x\in \underline{\left(F,A\right)\;\tilde{\cap }\;\left(G,B\right)}$.

(8) Let $x\in {\left(\bar{FA}\right)}^{c}\Rightarrow x\notin \bar{FA}\Rightarrow ∄\varepsilon \in A$ such that *x* ∈ *F*(*ε*)⇔ for all *ε* ∈ *A*, and $x\in {\left(F\left(\varepsilon \right)\right)}^{c}\Rightarrow x\in \underline{{\left(F\left(\varepsilon \right)\right)}^{c}}$.

(9)  $x\in \underline{{\left(F,A\right)}^{c}}\Rightarrow$ for all *ε* ∈ *A*,  *x* ∈ (*F*(*ε*))^*c*^⇒ for all *ε* ∈ *A*, and  $x\notin F\left(\varepsilon \right)\Rightarrow x\notin \underline{FA}\Rightarrow x\in {\left(\underline{FA}\right)}^{c}$.

The inclusions in (1, 3–6, 8–9) cannot be reversed in general, as it is shown in the following counterexamples.

**Example 12**

Let (*X*, *E*) be the soft space with *X* = {*a*, *b*, *c*, *d*, *e*} and *E* = {*e*_1_, *e*_2_, *e*_3_, *e*_4_}.

(3) For soft sets (*F*, *A*) = {*e*_1_ = {*a*, *b*, *c*, *d*},  *e*_2_ = {*a*, *c*},  *e*_3_ = {*a*, *b*, *e*}}, and (*G*, *B*) = {*e*_3_ = {*a*, *c*, *e*},  *e*_4_ = {*d*, *e*}}, we have

(13) $$\begin{array}{c} \bar{FA}\cup \bar{GB}=\left\{a,b,c,d,e\right\}⊈\left\{a,b,c,e\right\}=\bar{\left(F,A\right)⋓\left(G,B\right)}. \end{array}$$

(4) Choosing (*F*, *A*) = {*e*_2_ = {*c*, *d*, *e*},  *e*_3_ = {*a*, *b*, *e*}} and (*G*, *B*) = {*e*_2_ = {*c*, *d*},  *e*_3_ = {*b*, *d*},  *e*_4_ = {*d*, *e*}} gives

(14) $$\begin{array}{c} \bar{FA}\cap \bar{GB}=\left\{b,c,d,e\right\}⊈\left\{b,c,d\right\}=\bar{\left(F,A\right)⋒\left(G,B\right)}. \end{array}$$

(5) Choosing (*F*, *A*) = {*e*_1_ = {*a*, *b*, *c*, *d*, *e*},  *e*_2_ = {*b*, *d*, *e*},  *e*_4_ = {*a*, *b*, *d*}} and (*G*, *B*) = {*e*_1_ = {*e*},  *e*_2_ = {*c*, *e*}} gives

(15) $$\begin{array}{c} \underline{\left(F,A\right)⋓\left(G,B\right)}=\left\{b,c,d,e\right\}⊈\left\{b,d,e\right\}=\underline{FA}\cup \underline{GB}. \end{array}$$

(6) Choose (*F*, *A*) = {*e*_3_ = {*a*, *b*, *d*}} and (*G*, *B*) = {*e*_1_ = {*a*, *b*, *c*, *e*},  *e*_2_ = {*a*, *c*},  *e*_3_ = {*b*, *c*, *e*},  *e*_4_ = {*a*, *b*, *c*}}. By calculations we have

(16) $$\begin{array}{c} \underline{\left(F,A\right)⋒\left(G,B\right)}=\left\{b\right\}⊈\left\{\;\right\}=\underline{FA}\cap \underline{GB}. \end{array}$$

(8) Choose soft set (*F*, *A*) = {*e*_1_ = {*c*, *d*},  *e*_2_ = {*b*, *e*},  *e*_3_ = {*b*, *e*}}, then

(17) $$\begin{array}{c} \bar{{\left(F,A\right)}^{c}}=\left\{a,b,c,d,e\right\}⊈\left\{a\right\}={\left(\bar{FA}\right)}^{c}. \end{array}$$

(9) Choose soft set *FA* = {*e*_2_ = {*a*, *e*},  *e*_4_ = {*d*, *e*}}, then

(18) $$\begin{array}{c} {\left(\underline{FA}\right)}^{c}=\left\{a,b,c,d\right\}⊈\left\{b,c\right\}=\underline{{\left(F,A\right)}^{c}}. \end{array}$$

The membership relation is a basic issue when speaking about sets. In classical set theory, absolute knowledge is required about elements of the universe; that is, we assume that each element of the universe can be properly classified into a set *X* or into its complement. In soft set theory, this is not the case, since we claim that the membership relation is not a primitive notion but one that must be based on approximate descriptions we have. Consequently, three membership relations are necessary to express the fact that some objects of the universe cannot be properly classified employing the approximate descriptions. Thus, the notion of collection of approximate descriptions of an object leads to a new conception of membership relations which we define in the following.

**Definition 13**

For *x* ∈ *X* and a soft set (*F*, *A*) in soft space (*X*, *E*), we define “parameter set of $x\in \bar{FA}$” as

(19) $$\begin{array}{c} x_{\left(F,A\right)}=\left\{e\in A\;|\;x\in F\left(e\right)\right\}. \end{array}$$

Clearly *x*_(*F*,*A*)_⊆*A*⊆*E*.

**Definition 14**

For a soft set (*F*, *A*) the inverted soft set is denoted and defined as

(20) $$\begin{array}{c} {\left(F,A\right)}^{*}=\left\{x=x_{\left(F,A\right)}\;|\;x\in \bar{FA}\right\}. \end{array}$$

Clearly for (*F*, *A*) in soft space (*X*, *E*), the inverted soft set (*F*,*A*)* is in the soft space (*E*, *X*). We may call soft space (*E*, *X*) as an inversion of soft space (*X*, *E*). In the sequel, we may use F* and A* to denote the assignment function and set of parameters of the soft set (*F*,*A*)*; equivalently, we may also write (*F*,*A*)* = (F*, A*).

**Definition 15**

For a soft set (*F*, *A*) in soft space (*X*, *E*), we define three types of memberships as

1. $x\;\underline{\in }\;\left(F,A\right)$ if and only if $x\in \underline{FA}$,
2. $x\overset{n}{\in }\left(F,A\right)$ if and only if ||*x*_(*F*,*A*)_|| = *n*,
3. $x\;\bar{\in }\;\left(F,A\right)$ if and only if $x\in \bar{FA}$,

where $x\;\underline{\in }\;\left(F,A\right)$ reads “*x* is a core member of (*F*, *A*)” and $x\;\bar{\in }\;\left(F,A\right)$ reads “*x* is a support member of (*F*, *A*)” and will be called lower and upper membership relations, respectively.

**Proposition 16**

For soft sets (*F*, *A*) and (*G*, *B*) in a soft space (*X*, *E*), we have

1. $x\;\underline{\in }\;\left(F,A\right)\Leftrightarrow x\overset{||A||}{\in }\left(F,A\right)$,
2. $x\;\bar{\in }\;\left(F,A\right)\Leftrightarrow x\overset{n}{\in }\left(F,A\right)$,* where n* ≥ 1,
3. $x\overset{0}{\in }\left(F,A\right)\Leftrightarrow x\;\bar{\in }\;{\left(F,A\right)}^{c}$.

From Proposition 11 we immediately obtain the following properties of membership relations.

**Proposition 17**

For soft sets (*F*, *A*) and (*G*, *B*) in a soft space (*X*, *E*), we have:

1. $x\;\underline{\in }\;\left(F,A\right)$ $\Rightarrow x\;\bar{\in }\;\left(F,A\right)$,
2. (*F*, *A*)⊆(*G*, *B*)⇒$\left(x\;\bar{\in }\;\left(F,A\right)\Rightarrow x\;\bar{\in }\;\left(G,B\right)\right)$,
3. $x\;\bar{\in }\;\left(F,A\right)\cup \left(G,B\right)\Leftrightarrow x\;\bar{\in }\;\left(F,A\right)$ *or* $x\;\bar{\in }\;\left(G,B\right)$,
4. $x\;\bar{\in }\;\left(F,A\right)⋓\left(G,B\right)\Rightarrow x\;\bar{\in }\;\left(F,A\right)$ *or* $x\;\bar{\in }\;\left(G,B\right)$,
5. $x\;\bar{\in }\;\left(F,A\right)⋒\left(G,B\right)\Rightarrow x\;\bar{\in }\;\left(F,A\right)$ *and* $x\;\bar{\in }\;\left(G,B\right)$,
6. $x\;\underline{\in }\;\left(F,A\right)$ *or* $x\;\underline{\in }\;\left(G,B\right)\Rightarrow x\;\underline{\in }\;\left(F,A\right)⋓\left(G,B\right)$,
7. $x\;\underline{\in }\;\left(F,A\right)\cap \left(G,B\right)\Leftrightarrow x\;\underline{\in }\;\left(F,A\right)$ *and* $x\;\underline{\in }\;\left(G,B\right)$,
8. $x\;\underline{\in }\;\left(F,A\right)$ *and* $x\;\underline{\in }\;\left(G,B\right)\Rightarrow x\;\underline{\in }\;\left(F,A\right)⋒\left(G,B\right)$,
9. $x\;\underline{\in }\;\left(F,A\right)\cup \left(G,B\right)\Rightarrow x\;\bar{\in }\;\left(F,A\right)$ *or* $x\;\bar{\in }\;\left(G,B\right)$,
10. $x\;\underline{\in }\;\left(F,A\right)⋓\left(G,B\right)\Rightarrow x\;\bar{\in }\;\left(F,A\right)$ *or* $x\;\bar{\in }\;\left(G,B\right)$,
11. $x\;\underline{\in }\;\left(F,A\right)⋒\left(G,B\right)\Rightarrow x\;\bar{\in }\;\left(F,A\right)$ *and* $x\;\bar{\in }\;\left(G,B\right)$.

**Proof**

(2) Let $x\;\bar{\in }\;\left(F,A\right)$, and then ∃*ε* ∈ *A* such that *x* ∈ *F*(*ε*)⊆*G*(*ε*) (by hypothesis); hence, $x\;\bar{\in }\;\left(G,B\right)$.

(4)  $x\;\bar{\in }\;\left(F,A\right)⋓\left(G,B\right)\Rightarrow \exists \varepsilon \in A\cap B$ such that *x* ∈ *F*(*ε*) ∪ *G*(*ε*); that is, ∃*ε* ∈ *A* such that *x* ∈ *F*(*ε*) or ∃*ε* ∈ *B* such that *x* ∈ *G*(*ε*). Equivalently $x\;\bar{\in }\;\left(F,A\right)$ or $x\;\bar{\in }\;\left(G,B\right)$.

(5) It is similar to (4).

(6) Note that *A*∩*B* ≠ *ϕ*, as otherwise restricted union is not defined. Suppose $x\;\underline{\in }\;\left(G,B\right)$ only. Then for all *ε* ∈ *B*, *x* ∈ *G*(*ε*). As *A* and *B* are nondisjoint supposing *ε*′ ∈ *A*∩*B*, then *x* ∈ *F*(*ε*′) ∪ *G*(*ε*′). Since this holds for all *ε*′ ∈ *A*∩*B*, we have $x\;\underline{\in }\;\left(F,A\right)⋓\left(G,B\right)$.

In the following we give some relevant counterexamples.

**Example 18**

Let (*X*, *E*) be a soft space with *X* = {*a*, *b*, *c*, *d*, *e*} and *E* = {*e*_1_, *e*_2_, *e*_3_, *e*_4_}.

(4) Choose

(21) $$\begin{array}{c} \left(F,A\right)=\left\{e_{1}=\left\{b,d,e\right\},\;e_{3}=\left\{b,e\right\},\;e_{4}=\left\{b,c,d\right\}\right\},\\ \left(G,B\right)=\left\{e_{1}=\left\{a,b,e\right\}\right\}, \end{array}$$

and then $c\;\bar{\in }\;\left(F,A\right)$ but $c\;\bar{\notin }\;\left(F,A\right)⋓\left(G,B\right)$.

(5) Choose

(22) $$\begin{array}{c} \left(F,A\right)=\left\{e_{1}=\left\{a,d\right\},\;e_{4}=\left\{a,c,e\right\}\right\},\\ \left(G,B\right)=\left\{e_{2}=\left\{d,e\right\},\;e_{3}=\left\{\;\right\},\;e_{4}=\left\{a,b,c\right\}\right\}. \end{array}$$

Then $d\;\bar{\in }\;\left(F,A\right)$ and $d\;\bar{\in }\;\left(G,B\right)$ but $d\;\overset{-}{\notin }\;\left(F,A\right)⋒\left(G,B\right)$.

(6) Choose

(23) (F,A)={e1={ }, e3={a,c,d}},(G,B)={e1={a,d,e}, e2={a,c},e3={a,b}, e4={b,c,d,e}},

and then $d\;\underline{\in }\;\left(F,A\right)⋓\left(G,B\right)$ but $d\;\underline{\notin }\;\left(F,A\right)$ and $d\;\underline{\notin }\;\left(G,B\right)$.

(8) Choose

(24) $$\begin{array}{c} \left(F,A\right)=\left\{e_{3}=\left\{a,c,e\right\}\right\},\\ \left(G,B\right)=\left\{e_{1}=\left\{a,d\right\},\;e_{2}=\left\{a,b\right\},\;e_{3}=\left\{a,c,d,e\right\}\right\} \end{array}$$

then $c\;\underline{\in }\;\left(F,A\right)⋒\left(G,B\right)$ but $c\;\underline{\notin }\;\left(G,B\right)$.

(9) Choose

(25) $$\begin{array}{c} \left(F,A\right)=\left\{e_{2}=\left\{a,b\right\},\;e_{3}=\left\{c,d,e\right\},\;e_{4}=\left\{a,b,d\right\}\right\},\\ \left(G,B\right)=\left\{e_{2}=\left\{e,a,d\right\}\right\}, \end{array}$$

and then $c\;\bar{\in }\;\left(F,A\right)$ but $c\;\underline{\notin }\;\left(F,A\right)\cup \left(G,B\right)$.

(10) Choose

(26) (F,A)={e2={a,b,c}, e3={c}},(G,B)={e2={b,c,e}, e1={b,c},e3={a,b}, e4={a,b,c}},

and then $e\;\bar{\in }\;\left(G,B\right)$ but $e\;\underline{\notin }\;\left(F,A\right)⋓\left(G,B\right)$.

(11) Choose

(27) $$\begin{array}{c} \left(F,A\right)=\left\{e_{2}=\left\{b,c,d,e\right\},\;e_{3}=\left\{b,c,e\right\}\right\},\\ \left(G,B\right)=\left\{e_{1}=\left\{a,b,c,e\right\},\;e_{3}=\left\{a,d\right\},\;e_{4}=\left\{a,d\right\}\right\}, \end{array}$$

and then $c\;\bar{\in }\;\left(F,A\right)$ and $c\;\bar{\in }\;\left(G,B\right)$ but $c\;\underline{\notin }\;\left(F,A\right)⋒\left(G,B\right)$.

## 4. Upper and Lower Soft Approximations

**Definition 19**

For *S*⊆*X*, the induced soft set $\left(\tilde{S},A\right)$ in (*X*, *E*), where *A* ⊂ *E*, is denoted and defined as

(28) $$\begin{array}{c} {\tilde{S}}_{A}=\left\{e=S,\;\forall e\in A\right\}. \end{array}$$

This may be called “soft extension” of a subset *S* of *X* into the soft space (*X*, *E*). The induced soft set $\left(\tilde{S},E\right)$ is simply denoted as $\tilde{S}$. Obviously the null, full null, absolute, and full absolute soft sets are different soft extensions of *ϕ* and *X*, respectively, with suitable choices of *A*. We may call ${\tilde{S}}_{A}$, a constant soft set as well.

Using the above-defined notions of core, support, and constant soft set, we now define the upper and lower soft approximations as follows.

**Definition 20**

For a soft set (*F*, *A*) in soft space (*X*, *E*), we define

(29) $$\begin{array}{c} \underline{\left(F,A\right)}={\tilde{\underline{FA}}}_{A},\\ \bar{\left(F,A\right)}={\tilde{\bar{FA}}}_{A}. \end{array}$$

$\underline{\left(F,A\right)}$ and $\bar{\left(F,A\right)}$ are said to be lower and upper soft approximations of the soft set (*F*, *A*).

**Example 21**

Let (*F*, *A*) be a soft set in soft space (*X*, *E*), where *X* = {*x*_1_, *x*_2_, *x*_3_, *x*_4_} and *E* = {*e*_1_, *e*_2_, *e*_3_, *e*_4_} and

(30) $$\begin{array}{c} \left(F,A\right)=\left\{e_{1}=\left\{x_{1}\right\},\;e_{2}=\left\{x_{3},x_{4}\right\},\;e_{4}=\left\{x_{1},x_{4}\right\}\right\}. \end{array}$$

Then $\bar{FA}=\left\{x_{1},x_{3},x_{4}\right\}$ and $\underline{FA}=\left\{x_{1}\right\}$. Hence

(31) (F,A)¯=FA¯~A={e1={x1,x3,x4}, e2={x1,x3,x4}e4={x1,x3,x4}},(F,A)_=FA_~A={e1={x1}, e2={x1}, e4={x1}}.

The following results are immediate.

**Theorem 22**

For soft sets (*F*, *A*) and (*G*, *B*) in a soft space (*X*, *E*), we have

1. $\underline{\left(F,A\right)}\;\tilde{\subseteq }\;\left(F,A\right)\;\tilde{\subseteq }\;\bar{\left(F,A\right)}$,
2. $\bar{\left(F,A\right)\;\tilde{\cup }\;\left(G,B\right)}\;\tilde{\subseteq }\;\bar{\left(F,A\right)}\;\tilde{\cup }\;\bar{\left(G,B\right)}$,
3. $\underline{\left(F,A\right)\;\tilde{\cap }\;\left(G,B\right)}\;\tilde{=}\;\underline{\left(F,A\right)}\;\tilde{\cap }\;\underline{\left(G,B\right)}$,
4. $\bar{\neg \left(F,A\right)}=\bar{\left(F,A\right)}$,
5. $\underline{\neg \left(F,A\right)}=\underline{\left(F,A\right)}$,
6. $\left(F,A\right)\;\tilde{\subseteq }\;\left(G,B\right)\Rightarrow$ $\bar{\left(F,A\right)}\;\tilde{\subseteq }\;\bar{\left(G,B\right)}$.

### 4.1. An Application of Soft Approximation

Granular computing (GrC) is an umbrella term to cover any theories, methodologies, techniques, and tools that make use of granules in problem solving. The first appearance of the concept was in 1979 under the name of information granularity by L. A. Zadeh. Since then much research has been conducted in various aspects of granular computing. GrC has been studied by the help and applications of fuzzy set theory, rough set theory, and computing with Words. As Soft Set Theory is a general case of both FST and RST, there has been natural tendency to explore SST on lines similar to RST and FST. One such effort is the work of Feng et al. (2010) [8]. Using a soft set, the authors granulate the universe of discourse, a classical set, as follows.

**Definition 23**

Let *𝒢* = (*f*, *A*) be a soft set over *U*. Then the pair *P* = (*U*, *𝒢*) is called a soft approximation space. Based on *P*, we define the following two operations:

(32) $$\begin{array}{c} {\underline{\text{apr}}}_{P}\left(X\right)=\left\{u\in U:\exists a\in A\left[u\in f\left(a\right)\subseteq X\right]\right\},\\ {\bar{\text{apr}}}_{P}\left(X\right)=\left\{u\in U:\exists a\in A\left[u\in f\left(a\right),\;f\left(a\right)\cap X\neq \phi \right]\right\}, \end{array}$$

assigning to every subset *X*⊆*U* two sets ${\underline{\text{apr}}}_{P}\left(X\right)$ and ${\bar{\text{apr}}}_{P}\left(X\right)$ called the lower and upper soft rough approximations of *X* in *P*, respectively.

**Remark 24**

Note that

(33) $$\begin{array}{c} {\underline{\text{apr}}}_{P}\left(X\right)\subseteq \bar{FA},\\ {\bar{\text{apr}}}_{P}\left(X\right)\subseteq \underline{FA}. \end{array}$$

A more interesting possibility is that of granulation of soft space itself instead of just the universe of discourse. This direction may open very promising prospects for Soft Set Theory. Making such an attempt in the following we show how the notion of soft approximation may be used for granulation of soft space.

**Definition 25**

Soft sets (*F*, *A*) and (*G*, *B*) in a soft space (*X*, *E*) are said to be g-related (read as “granule related”) if

(34) $$\begin{array}{c} \bar{FA}=\bar{GB},\;\;\underline{FA}=\underline{GB}. \end{array}$$

It is denoted as (*F*, *A*)≑(*G*, *B*).

**Definition 26**

For a soft set (*F*, *A*) in soft space (*X*, *E*), the class of all g-related soft sets is denoted as 〈(*F*, *A*)〉. Symbolically

(35) $$\begin{array}{c} \langle \left(F,A\right)\rangle =\left\{\left(G,B\right)\;|\;\left(G,B\right)≑\left(F,A\right)\right\}. \end{array}$$

Besides an investigation into the nature of the abovementioned granulation of soft space, it would also be a very interesting direction of research to investigate the granulation of a soft space (*X*, *E*) and its relation with the granulation of inverted soft space (*E*, *X*) for a given soft set (*F*, *A*). Such a granulation of inverted soft space may be sketched as

(36) $$\begin{array}{c} {\langle \left(F,A\right)\rangle }^{*}=\left\{\left(H,C\right)\in \left(E,X\right)\;|\;\left(H,C\right)≑{\left(F,A\right)}^{*}\right\}. \end{array}$$

While this direction of investigation is under our study now, results from other researchers are most eagerly awaited.

We now present the main result of this work: proving that the uni-int expression [14] is equivalent to a much simpler expression comprising only core and support of soft sets. For this we have the following.

**Theorem 27**

For soft sets we have

- $uni_{x}int_{y}\left(F_{A},G_{B}\right)=\bar{FA}\cap \underline{GB}$,
- $uni_{y}int_{x}\left(F_{A},G_{B}\right)=\bar{GB}\cap \underline{FA}$,
- $uni\text{-}int\left(F_{A},G_{B}\right)=\left[\bar{FA}\cap \underline{GB}\right]\cup \left[\bar{GB}\cap \underline{FA}\right]$.

**Proof**

(i) Suppose

(37) u∈⋃x∈A(⋂y∈BfA∧B(x,y))⟺u∈⋂y∈BfA∧B(x,y) for  some  x∈A⟺u∈fA∧B(x,y) ∀y∈B⟺u∈fA∧B(x,y) for  some  x∈A,  ∀y∈B⟺u∈fA(x) for  some  x∈A,  u∈fB(y)  ∀y∈B⟺u∈FA¯,  u∈GB_⟺u∈FA¯∩GB_.

(ii) It is similar to (i).

(iii) It follows directly from definition and (i) and (ii).

By optimum choice we mean the following.

**Definition 28**

For two soft sets (*F*, *A*) and (*G*, *B*) in soft space (*X*, *E*), an element *x* ∈ *X* is said to be an optimum choice if both ||*x*_(*F*,*A*)_|| and ||*x*_(*G*,*B*)_|| are simultaneously as larger as possible.

A possible improvement of the uni-int method may be given as

(38) $$\begin{array}{c} \text{optimum-choice}=\text{un}{\text{i}}_{x}\text{in}{\text{t}}_{y}\left(F_{A},G_{B}\right)\cap \text{un}{\text{i}}_{y}\text{in}{\text{t}}_{x}\left(F_{A},G_{B}\right). \end{array}$$

Such an improvement in the uni-int method still does not set this method to be fully valid. For example if the sets uni_*x*_int⁡_*y*_ and uni_*y*_int⁡_*x*_ are disjoint, the improved uni-int method fails to furnish a result. Such a case is in fact more frequently occurring in real decision making environments, where seldom an object fulfills all the required attributes. We illustrate this in the following.

**Example 29**

Suppose that a married couple, Mr. X and Mrs. X, come to the real estate agent to buy a house. If each partner has to consider their own set of parameters, then we select a house on the basis of the sets of partners. First, Mr. X and Mrs. X have to choose the sets of their parameters, *A* = {*e*_2_, *e*_3_, *e*_4_} and *B* = {*e*_1_, *e*_3_, *e*_4_}, respectively. We can now write the following soft sets depending upon the parameters of each Mr. X and Mrs. X:

(39) (F,A)={e2={u2,u3,u4}, e3={u4,u5},e4={u1,u2,u4,u5}},(G,B)={e1={u2,u3,u4}, e3={u3,u5},e4={u1,u2,u3}}.

Then the calculations show that

(40) $$\begin{array}{c} {\text{uni}}_{x}{\mathrm{int}}_{y}\left(F_{A},G_{B}\right)=\left\{u_{3}\right\},\;\;{\text{uni}}_{y}{\mathrm{int}}_{x}\left(F_{A},G_{B}\right)=\left\{u_{4}\right\}. \end{array}$$

Hence,

(41) uni-int(FA,GB)=unixint⁡y(FA,GB)∩uniyint⁡x(FA,GB)=ϕ.

Hence Cagman's uni-int gives incorrect decision and the improved uni-int does not yield any result. But a close examination of the soft sets (*F*, *A*) and (*G*, *B*) reveals that the house *u*_2_ is the one which fulfills maximum attributes of both Mr. X and Mrs. X.  Table 1 shows the analysis.

It is also noteworthy that the attribute *e*_4_ is shared by both Mr. X and Mrs. X. Consequently, in such a case *u*_2_ should be chosen to be the optimum choice (cf.  Definition 28).

In view of all the points raised in our above comments, we propose the following.

**Conjecture 30**

For soft sets (*F*, *A*) and (*G*, *B*) in a soft space (*X*, *E*), the optimum choice is given as

(42) {u∈C∗ ∣ H∗(u)≠ϕ, ||F∗(u)||||G∗(u)||−||F∗(u)||−||G∗(u)|| is  maximum},

where (*H*,*C*)* = (*F*,*A*)*⋒(*G*,*B*)*.

The conjecture requires that there should be at least one common attribute for both parties; for otherwise, it would be an unrealistic problem. The expression (*F*,*A*)*⋒(*G*,*B*)* ensures that the common attributes are considered on priority. The expression ||F*(*u*)||||G*(*u*)|| − ||F*(*u*)|| − ||G*(*u*)|| is meant to satisfy as many attributes of both parties as possible, while treating each one at par. This conjecture is applicable to the class of decision problems where maximum possible requirements of two parties are to be satisfied simultaneously (cf.  Definition 28).

In the following we give a detailed example to illustrate Conjecture 30.

**Example 31**

Assume that a real estate agent has a set of different types of houses *U* = {*u*_1_, *u*_2_, *u*_3_, *u*_4_, *u*_5_} which may be characterized by a set of parameters *E* = {*e*_1_, *e*_2_, *e*_3_, *e*_4_, *e*_5_, *e*_6_, *e*_7_}. For *i* = 1,…, 7 the parameters *e*_*i*_ stand for “in good location,” “cheap,” “modern,” “large,” “wooden,” “beautiful,” and “wide,” respectively. Suppose that this time another married couple, Mr. Y and Mrs. Y, come to the real estate agent to buy a house. If each partner has to consider their own set of parameters, then we select a house on the basis of the sets of partners. First, Mr. Y and Mrs. Y have to choose the sets of their parameters, *A* = {*e*_1_, *e*_4_, *e*_5_, *e*_6_, *e*_7_} and *B* = {*e*_2_, *e*_3_, *e*_4_, *e*_5_, *e*_6_, *e*_7_}, respectively. We can now write the following soft sets depending upon the parameters of each Mr. Y and Mrs. Y:

(43) (F,A)={e1={u1,u3,u4,u5}, e4={u4},e5={u2}, e6={u3}, e7={u3}},(G,B)={e2={u2,u3,u5}, e3={u2,u3,u5},e4={u1,u2}, e5={u2,u3,u4},e6={u1,u2,u4,u5}, e7={u3,u4}}.

Using uni-int method of Cagman we get

(44) $$\begin{array}{c} {\text{uni}}_{x}{\mathrm{int}}_{y}=\left\{\;\right\},\;\;{\text{uni}}_{y}{\mathrm{int}}_{x}=\left\{\;\right\},\\ \text{uni-}{\mathrm{int}}_{\text{Cagman}}=\left\{\;\right\},\;\;\text{uni-}{\mathrm{int}}_{\text{improved}}=\left\{\;\right\}. \end{array}$$

Clearly, both uni-int methods, that is, Cagman's and the improved one do not yield any result here. We now solve this using Conjecture 30 as follows:

(45) (F,A)∗={u1={e1}, u2={e5}, u3={e1,e6,e7},u4={e1,e4}, u5={e1}},(G,B)∗={u1={e4,e6}, u2={e2,e3,e4,e5,e6},u3={e2,e3,e5,e7}, u4={e5,e6,e7},u5={e2,e3,e6}},(H,C)∗=(F,A)∗⋒(G,B)∗={u1={ },u2={e5},u3={e7},u4={ },u5={ }}.

Calculating the decision value for each *u* ∈ C* such that H*(*u*) ≠ *ϕ*, we get

(46) $$\begin{array}{c} \left(u_{2},-3\right),\;\;\left(u_{3},2\right). \end{array}$$

Clearly optimum choice is *u*_3_ as it carries the maximum decision value of 2. The choice of *u*_3_ as optimum choice is justified as it satisfies one consensus attribute, namely, *e*_7_, three attributes of (*F*, *A*), and four attributes of (*G*, *B*). Thus *u*_3_ is the optimum choice in view of Definition 28.  Table 2 makes the claim more clear.

## 5. Conclusion

Notions of core and support of a soft set have been defined, and their fundamental properties studied. Core and support permit us to introduce and study three different notions of membership. Two more notions of very interesting characteristics are “soft set induced by a subset of *X*” and “inverted soft set.” These notions, in turn, define upper and lower soft approximations of a soft set. Such soft approximations are shown to have links with upper and lower approximations of a subset of universe introduced by Feng et al. (2010) in [8]. Whereas, Feng et al. try to granulate only the universe of discourse, we are interested in granulation of the soft space itself. One possible answer is provided by the help of soft approximations.

We present a new conjecture for finding the optimum choice between two parties. Our Example 31 presents a case where the new conjecture solves the problem correctly.

## Figures and Tables

**Table 1:** 

House	Attributes fulfilled	
	Mr. X	Mrs. X
*u*_2_	*e*_2_, *e*_4_	*e*_1_, *e*_4_
*u*_3_	*e*_3_	*e*_1_, *e*_3_, *e*_4_
*u*_4_	*e*_2_, *e*_3_, *e*_4_	*e*_1_

**Table 2:** 

House	Mr. Y		Mrs. Y	
	Attrib.	Count	Attrib.	Count
*u*_1_	*e*_1_	1	*e*_4_, *e*_6_	2
*u*_2_	*e*_5_	1	*e*_2_, *e*_3_, *e*_4_, *e*_5_, *e*_6_	5
*u*_3_	*e*_1_, *e*_6_, *e*_7_	3	*e*_2_, *e*_3_, *e*_5_, *e*_7_	4
*u*_4_	*e*_1_, *e*_4_	2	*e*_5_, *e*_6_, *e*_7_	3
*u*_5_	*e*_1_	1	*e*_2_, *e*_3_, *e*_6_	3
